# Donor Muse Cell Treatment Without HLA-Matching Tests and Immunosuppressant Treatment

**DOI:** 10.1093/stcltm/szae018

**Published:** 2024-06-10

**Authors:** Shinya Minatoguchi, Yasuyuki Fujita, Kuniyasu Niizuma, Teiji Tominaga, Toru Yamashita, Koji Abe, Mari Dezawa

**Affiliations:** Department of Cardiology, Gifu Municipal Hospital, Gifu, Japan; Department of Dermatology, Sapporo City General Hospital, Sapporo, Japan; Department of Neurosurgical Engineering and Translational Neuroscience, Graduate School of Biomedical Engineering, Tohoku University, Sendai, Japan; Department of Neurosurgical Engineering and Translational Neuroscience, Tohoku University Graduate School of Medicine, Sendai, Japan; Department of Neurosurgery, Tohoku University Graduate School of Medicine, Sendai, Japan; Research Division of Muse Cell Clinical Research, Tohoku University Hospital, Sendai, Japan; Department of Neurosurgery, Tohoku University Graduate School of Medicine, Sendai, Japan; Department of Neurology, Okayama University Graduate School of Medicine, Dentistry and Pharmaceutical Sciences, Okayama, Japan; National Center of Neurology and Psychiatry, Kodaira, Tokyo; Department of Stem Cell Biology and Histology, Tohoku University Graduate School of Medicine, Sendai, Japan

**Keywords:** pluripotent, sphingosine-1-phosphate, intravenous injection, immunotolerance, myocardial infarction, epidermolysis bullosa, amyotrophic lateral sclerosis, stroke

## Abstract

The strength of stem cell therapy is the regeneration of tissues by synergistic pleiotropic effects. Among many stem cell types, mesenchymal stem cells (MSCs) that are comprised of heterogenous population are widely used for clinical applications with the expectation of pleiotropic bystander effects. Muse cells are pluripotent-like/macrophage-like stem cells distributed in the bone marrow, peripheral blood, and organ connective tissues as cells positive for the pluripotent surface marker stage-specific-embryonic antigen -3. Muse cells comprise ~1% to several percent of MSCs. While Muse cells and MSCs share several characteristics, such as mesenchymal surface marker expression and their bystander effects, Muse cells exhibit unique characteristics not observed in MSCs. These unique characteristics of Muse cells include selective homing to damaged tissue after intravenous injection rather than being trapped in the lung like MSCs, replacement of a wide range of damaged/apoptotic cells by differentiation through phagocytosis, and long-lasting immunotolerance for donor cell use. In this review, we focus on the basic properties of Muse cells clarified through preclinical studies and clinical trials conducted by intravenous injection of donor-Muse cells without HLA-matching tests or immunosuppressant treatment. MSCs are considered to differentiate into osteogenic, chondrogenic, and adipogenic cells, whereas the range of their differentiation has long been debated. Muse cells may provide clues to the wide-ranging differentiation potential of MSCs that are observed with low frequency. Furthermore, the utilization of Muse cells may provide a novel strategy for clinical treatment.

## Graphical abstract

**Graphical Abstract:**
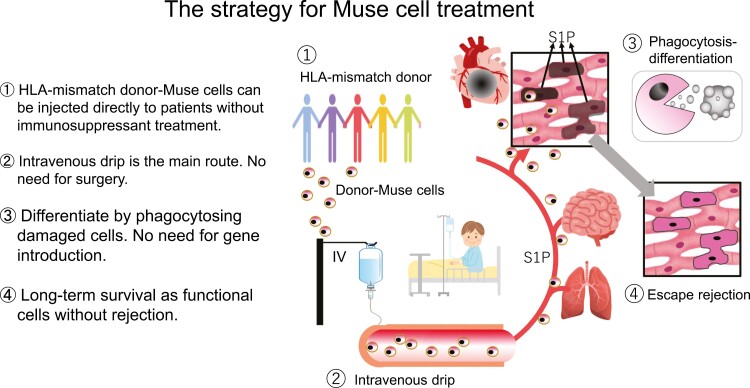

**Significance Statement**

MSCs differentiate into osteogenic, chondrogenic, and adipogenic cells, whereas their differentiation across germ layers is debated. Muse cells are pluripotent-like stem cells widely distributed in the body and comprise ~1% to several percent of MSCs. They selectively home to damaged sites after intravenous injection and replace apoptotic cells by differentiating into a variety of cell types by recycling signals derived from the phagocytosed apoptotic cells. This review summarizes the basic characteristics of muse cells and discusses their potential therapeutic efficacy, focusing on preclinical and clinical effects on acute myocardial infarction, epidermolysis bullosa, stroke, and ALS.

## Introduction

Drug development contributes to solving difficult-to-treat diseases and has delivered great benefits for a variety of diseases, including cancer and infectious and inflammatory diseases. In tissue degenerative diseases where cells are damaged, die, or lose their function, however, drugs cannot replace lost cells. Cell therapy using stem cells is expected to be a novel therapeutic strategy that may overcome this problem. While various types of stem cells have been tested in preclinical studies, somatic stem cells that can be isolated from the living body, particularly from adults, have low risks of tumorigenicity and are thus the main cell types used for clinical applications. Among these, mesenchymal stem cells (MSCs) are one of the generally used stem cell types because they are obtainable from easily accessible sources such as the bone marrow (BM), umbilical cord, and adipose tissue, and are expandable to a clinical scale.^1^

Cell therapy is expected to have various effects, such as pleiotropic bystander effects in which tissues are protected through cytokine production, activation of endogenous tissue stem cells/progenitors, vascular protection, immune modulation, neovascularization, and inflammation-suppression, activities that synergistically contribute to creating a microenvironment supportive of tissue repair.^1,2^ MSCs are a heterogeneous population comprised of mesenchymal cells and other types of cells, and are usually collected as adherent cells that rescue damaged tissue mainly by pleiotropic bystander effects.^1-3^ Multilineage-differentiating stress enduring (Muse) cells are a specific subpopulation of MSCs that corresponds to ~1% to several percent of the total MSC population.^4^ They are identified as cells positive for the pluripotent surface marker stage-specific-embryonic antigen (SSEA)-3 in the BM, adipose tissue, umbilical cord, and dermis, as well as in the peripheral blood and connective tissues of various organs.^4-8^ They are also present as ~1% to several percent of cultured fibroblasts.^9^ Muse cells have several characteristics in common with MSCs, such as the expression of mesenchymal surface markers and the pleiotropic bystander effects mentioned above.^2^ Muse cells also exhibit several unique characteristics not observed in MSCs. They selectively migrate to damaged tissue after intravenous injection rather than being trapped in the lung like MSCs, replace damaged/apoptotic cells by in vivo-differentiation through phagocytosing damaged/apoptotic cells, and repair damaged tissue.^2^

MSCs are known to differentiate into osteogenic, chondrogenic, and adipogenic cells, but their wide-ranging pluripotent-like differentiation, such as into neural, cardiac, and hepatic lineages, has been debated.^2,10,11^ This might be partly due to the heterogeneity of MSCs, and the low-frequency occurrence of differentiation across the triploblastic-lineage boundaries. Muse cells might provide clues to the “differentiability” of MSCs. In this review, we describe the unique characteristics of Muse cells clarified through preclinical and clinical studies and discuss their potential application for cell therapy.

## Primary Characteristics of Muse Cells

Muse cells, first discovered as a stress-tolerant subpopulation of BM-MSCs, are now known to be endogenous reparative stem cells with both pluripotent-like and macrophage-like properties.^12^ They are identified as cells positive for the pluripotent surface marker stage-specific embryonic antigen (SSEA)-3 in the BM (0.01%-0.03% of the mononuclear fraction), peripheral blood (0.01%-0.2% of the mononuclear fraction), and connective tissues of various organs, including the umbilical cord and amnion.^5,7,13,14^ Consistent with their being endogenous to the body, Muse cells exhibit low telomerase activity and do not form teratomas after transplantation in vivo.^9,14^ They comprise ~1% to several percent of the total population of MSCs and fibroblasts, expressing both SSEA-3 and mesenchymal markers (ie, CD29, CD44, CD90,CD73, and CD105).^4,9^ As their doubling time is ~1.3 days per cell division, comparable to that of fibroblasts, they can be grown at clinically relevant scales.^4,9^

**Figure 1.**
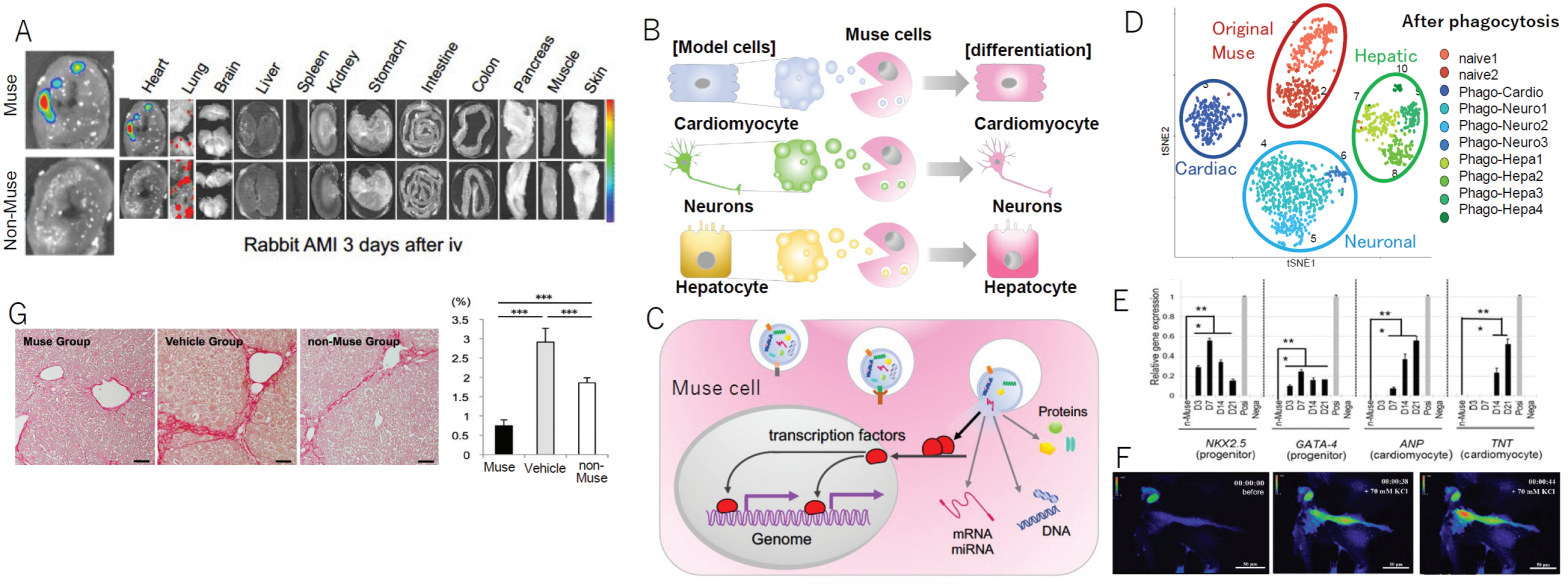
Muse cell characteristics. (**A**) Muse cells selectively migrate to the site of damage following intravenous administration. A rabbit AMI model 3 days after intravenous injection of nano-lantern-Muse and non-Muse cells exhibited specific homing of Muse cells to the post-infarct heart tissue, while non-Muse cells were trapped in the lung and rarely homed to the heart.^15^ (**B**) Differentiation of Muse cells into triploblastic-lineage cells by phagocytosis. (**C**) The mechanism underlying how Muse cells recycle signals from the up-taken damaged/apoptotic cells necessary for differentiation, such as transcription factors. (**D**) Single-cell RNA sequencing of human Muse cells (original Muse cells) after phagocytosing apoptotic cell fragments of mouse hepatic- (hepatic), mouse cardiac- (cardiac), and rat neural- (neuronal) cells (7 days). Gene expression in differentiated Muse cells is distinct and differs from that in original Muse cells.^12^ (**E**) Cardiac marker expression in quantitative PCR in human Muse cells after phagocytosing apoptotic mouse cardiac cell fragments.^12^ (**F**) Functional assessment of Muse cell-derived cardiac cells after phagocytosis. Intracellular calcium dynamics in green fluorescent protein (GFP)-based Ca calmodulin probe (GCaMP)-h-Muse cells after biochemical depolarization with 70 mM KCl.^12^ (**G**) Evaluation of fibrosis by Sirius red staining in a mouse liver chronic fibrosis model with intravenous injection of human Muse cells, -non-Muse cells, or vehicle at 8 weeks. Anti-fibrosis effect was prominent in Muse cells.^80^ Figures were redrawn from Yamada et al,^15^ Wakao et al,^12^ and Iseki et al^80^ with permission.

Muse cells are pluripotent-like; they express pluripotency genes such as Nanog, Oct3/4, and Sox2 at moderate levels compared with embryonic stem and induced pluripotent stem cells,^4,9^ and exhibit triploblastic-lineage differentiation at a single-cell level that is self-renewable over generations.^4^ Muse cells are macrophage-like; they selectively migrate to sites of damage in the body by sensing sphingosine-1-phosphate (S1P) produced by damaged/apoptotic cells, and engulf the damaged cells.^12,15^ The responsiveness to S1P in Muse cells driven by S1P receptor 2 is swift; intravenously administered Muse cells selectively accumulate at the post-infarct area by day 1 in a lacuna stroke model and by day 3 in an acute myocardial infarction (AMI) model (Fig. 1A).^15,16^ Unlike macrophages, however, muse cells directly recycle signals from the phagocytosed damaged/apoptotic cells that are necessary for differentiation, such as transcription factors, and quickly differentiate into the same cell type as the damaged/apoptotic cell within days with few errors (Fig. 1B–1F).^12^ There are several hypotheses regarding the differentiation mechanism. One of the possible mechanisms is that the factors necessary for differentiation, such as transcription factors, translocate into the Muse cell nucleus and bind to promoter regions of the genome, leading to differentiation into the same cell type as the phagocytosed-damaged/apoptotic cell (Fig. 1B–1F).^12^ In any case, differentiation progressed rapidly on a daily basis.^9^ In this manner, Muse cells might be able to replace various types of damaged/apoptotic cells with healthy functioning cells in the tissue because they are pluripotent-like. Simultaneously, Muse cells participate in neovascularization, which is essential for tissue repair and maintenance, by differentiating into vascular cells, as demonstrated in many tissue damage models.^15,17,18^

In addition to cell replacement, like MSCs, Muse cells exhibit bystander effects, such as anti-inflammatory, anti-fibrosis, anti-apoptotic, and tissue protection effects (Fig. 1G).^2^ They secrete various cytokines such as interleukin (IL)-10, transforming growth factor-β (TGF-β), prostaglandin E2 (PGE2), vascular endothelial growth factor, platelet-derived growth factor, fibroblast growth factor, and hepatocyte growth factor (HGF).^17,19^ Muse cells also produce matrix metalloproteases-1 (MMP1), MMP2, and MMP9, which are known to suppress fibrosis.^15^

Overall, Muse cells are considered to repair tissues by synergistic mechanisms of rapid and selective homing to damaged sites, cell replacement by differentiation, and bystander effects.

Clinical data suggest that Muse cells are involved in endogenous reparative activity. The dynamics of Muse cells in the peripheral blood of patients with ischemic stroke, AMI, and liver surgery indicate that (1) the peripheral blood-Muse cell number increases following an increase in serum S1P, (2) Muse cells exhibit 3 patterns of change after onset: increase, decrease, and no change, and (3) patients with a higher peripheral blood-Muse cell number show better recovery.^8,20,21^ In an AMI study, patients with a higher number of peripheral blood-Muse cells in the acute phase (by day 7 after onset) exhibited statistically meaningful recovery of cardiac function with a lower occurrence of heart failure in the chronic stage (6 months after onset) compared to patients with no increase in the number of peripheral blood-Muse cells during the acute phase, suggesting the involvement of Muse cells in the innate reparative function.^8^

**Figure 2.**
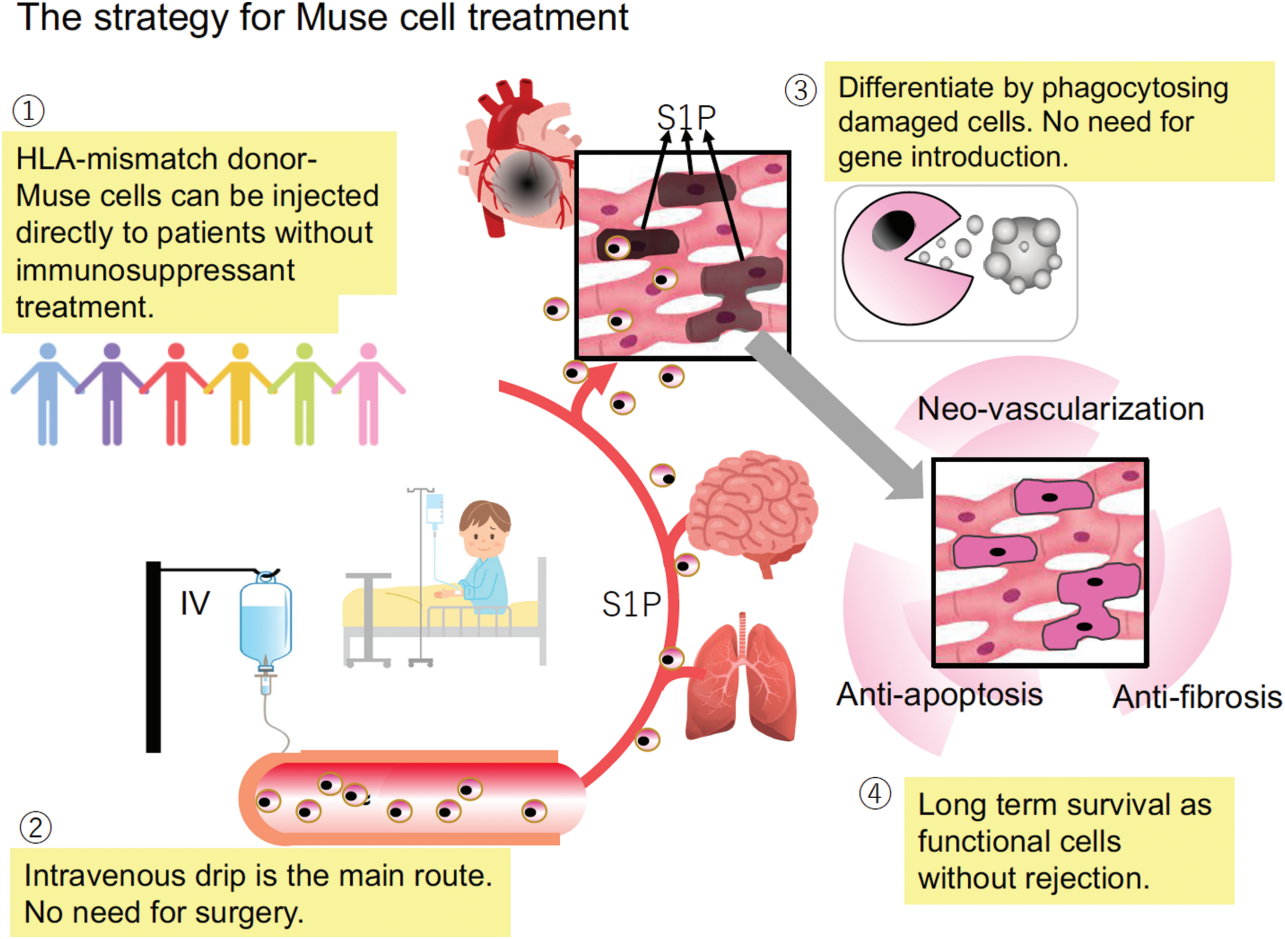
Strategy for Muse cell treatment. Muse cells have several outstanding characteristics beneficial for clinical application; (1) HLA-mismatched donor Muse cells can be directly administered to patients without immunosuppressant, due to specific immunotolerance, (2) because Muse cells selectively migrate to damage sites by S1P-S1P receptor 2 axis, intravenous drip is a more efficient method of delivering them to the damaged site than a surgical approach, (3) unlike embryonic stem and induced pluripotent stem cells, Muse cells do not require differentiation induction because they can differentiate into the same cell type as the damaged/apoptotic cell by phagocytosis to replace damaged/apoptotic cells with healthy functioning cells, (4) Muse cells remain in the homed tissue for an extended period without rejection, and thus their bystander effects such as anti-apoptotic and anti-fibrosis effects as well as neovascularization are long lasting.

In addition to the above characteristics, Muse cells have beneficial properties for clinical applications (Fig. 2). While HLA matching tests and long-term immunosuppression are generally required for organ and BM transplantation, HLA-mismatched donor Muse cells can be directly administered to patients without long-term immunosuppressant treatment and can survive in the host tissue for an extended period without immune rejection. Although the full details of their immunotolerance have not yet been elucidated, the underlying mechanism is partly explained by the expression of HLA-G, a key factor for immune tolerance in the placenta, as well as the production of indoleamine 2, 3-dioxygenase, TGF-β, PGE2, nitric oxide, and HGF, which are related to regulatory T-cell activation, suppression of T-cell proliferation, and antigen-presenting dendritic cell differentiation.^2,15,17^ In animal models, allogeneic-Muse cells (rabbit AMI and swine hepatectomy models)^15,22^ and xenogeneic-Muse cells (human Muse cells injected into mouse chronic kidney, rabbit AMI, mouse epidermolysis bullosa (EB), rat perinatal hypoxic-ischemic encephalopathy, and rat interstitial cystitis models)^15,17,18,23,24^ escaped immune rejection and survived in the host tissue as integrated functional cells for an extended period without immunosupressant treatment.

Taking advantage of these unique characteristics, the strategy for Muse cell treatment is as follows:

1. HLA-mismatch donor Muse cells can be administered directly without immunosuppression.
2. Intravenous drip is the central administration route and surgery is not required to deliver Muse cells to the target tissue.
3. Gene manipulations to render pluripotency are unnecessary because Muse cells have triploblastic-lineage differentiation ability.
4. Cytokine treatment for differentiation induction before treatment is not necessary because Muse cells replace damaged/apoptotic cells by phagocytosis and differentiation with few errors in vivo.
5. The anti-inflammatory effects of Muse cell treatment are long-lasting because Muse cells, even allogeneic Muse cells, survive in the host tissue as integrated cells for an extended period.
6. The stress tolerance and low risk of tumorigenesis of Muse cells are beneficial for application to diseases with tissue destruction (Fig. 2).

Clinical trials for AMI, ischemic stroke, EB, amyotrophic lateral sclerosis (ALS), cervical spinal cord injury, neonatal hypoxic-ischemic encephalopathy, and COVID-19 respiratory distress syndrome have been conducted by intravenous administration of donor-derived Muse cells without HLA-matching test or immunosupressant treatment. In the following sections, we focus on the functional characteristics of Muse cells clarified through preclinical and clinical studies on AMI, EB, ischemic stroke, and ALS.^25-28^

To compare the outcome between Muse cells and MSCs, some preclinical studies evaluated Muse and MSC groups, while other studies critically separated Muse and non-Muse cells from BM-MSCs as SSEA-3-positive and SSEA-3-negative fractions and administered these fractions to animal models to evaluate Muse and non-Muse groups.^9^ Clinical-grade allogeneic-human BM-Muse cell product CL2020 produced by Life Science Institute Inc. (Tokyo, Japan), a Mitsubishi Chemical Holdings Corporation group company, was used in the clinical trials. Each clinical trial was approved by the Clinical Trial Review Committee in the hospital and conducted in compliance with the Japanese “Act on Securing Quality, Efficacy, and Safety of Products Including Pharmaceuticals and Medical Devices,” the “Ordinance on Good Clinical Practice of Regenerative Medical Products, etc.,” and the Declaration of Helsinki. Patients (or family members, if required) provided written informed consent.

## Acute Myocardial Infarction (AMI)

### Cell Therapy for AMI

Acute myocardial infarction is a leading cause of morbidity and mortality worldwide.^29^ The current first-line therapy for AMI is reperfusion of the occluded coronary artery by percutaneous coronary intervention (PCI) at the earliest opportunity after onset. Despite the efficacy of PCI therapy, AMI sometimes causes a significant loss of cardiomyocytes due to occlusion of the main coronary arteries, late timing of the procedure, or failure of reperfusion, leading to left ventricular (LV) dysfunction and LV remodeling. An ST-elevation myocardial infarction (STEMI) in patients with LV ejection fraction (LVEF) ≤ 45% after successful PCI in the acute phase is associated with a lower rate of significant adverse cardiac event-free survival compared to patients with LVEF > 45%.^30,31^ Therefore, the degree of LVEF recovery in the acute phase predicts the long-term prognosis.^32^ Indeed, LV dilation, so-called LV remodeling, at 6 months is associated with poor long-term clinical outcomes.^33^ Therefore, better recovery of LV function and attenuation of LV dilation during the acute phase indicate a better prognosis and stem cell therapy is a potential therapeutic strategy for treating AMI.

In previous preclinical studies, BM-mononuclear cells (MNCs) and MSCs were the major cell types infused into AMI models utilizing rodents, rabbits, swine, and other species, either by intramyocardial, intracoronary, or intravenous injection. Beneficial effects, such as the recovery of LVEF, were observed in many cases.^34^

In clinical trials, as well, BM-MNCs and MSCs were the major cell types administered to AMI patients by intracoronary, intravenous, or intramyocardial injection. The follow-up period was ~4 months to several years.^35^ Due to variations in the sample size, administration timing, administration method, follow-up period, and number of cells administered, the outcome of each trial cannot be compared. Many of the trials, however, reported therapeutic effects represented by the recovery in LVEF and a reduction of the infarct size.^35^ In these preclinical and clinical studies, the therapeutic mechanism of those cells was mainly attributed to bystander effects, while the contribution of other unknown mechanisms remains unclear.^34,35^ Muse cells exhibit characteristics similar to those of MSCs while they also exhibit characteristics distinct from those of MSCs. In the following section, we describe the mechanism of how Muse cells deliver therapeutic effects in AMI in preclinical and clinical studies and compare the mechanism with that of non-Muse cells and MSCs.

### Preclinical Studies of Muse cells in Rabbit and Mini-Pig AMI Models

**Figure 3.**
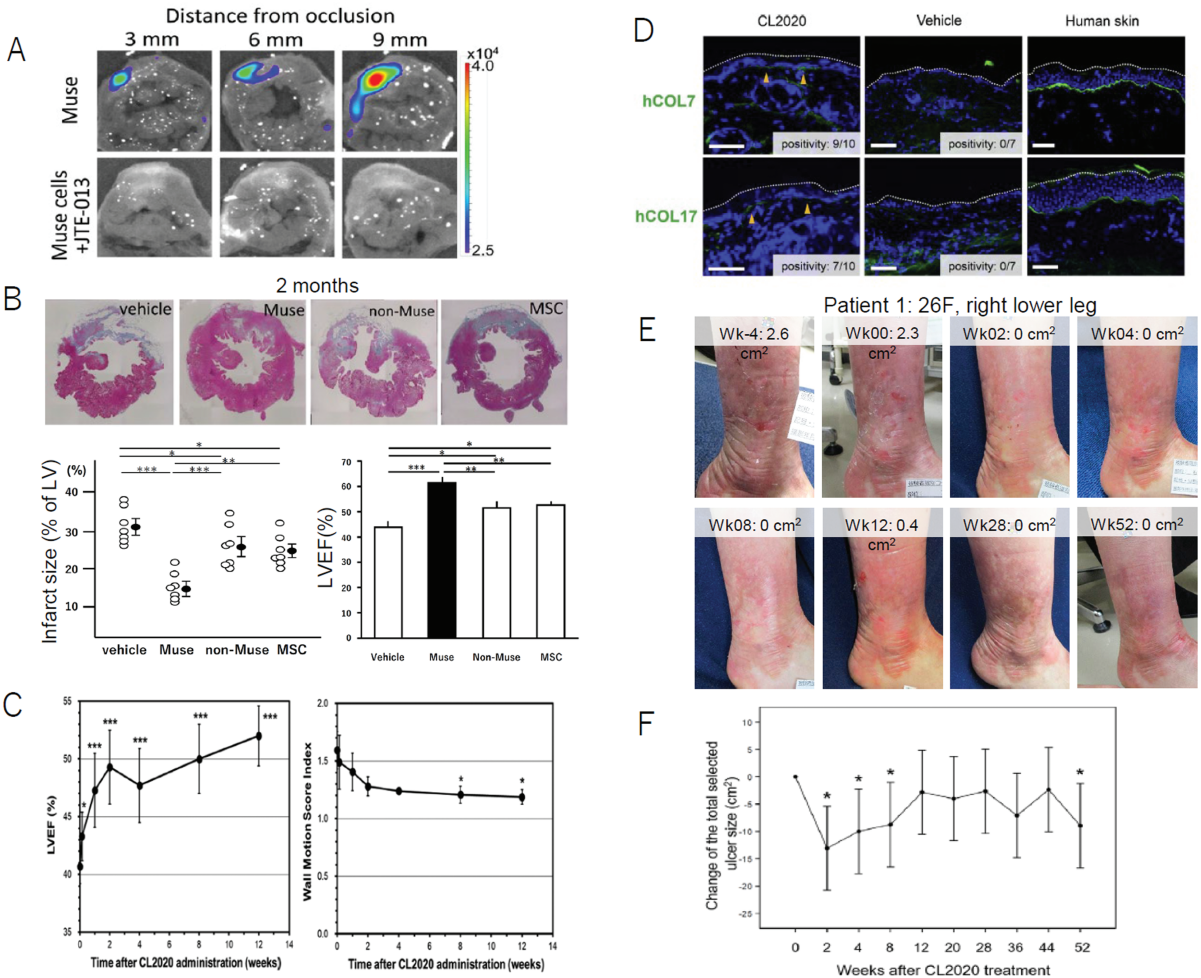
Preclinical and clinical trials in AMI and EB. (**A-B**) AMI rabbit model that received intravenous injection of Muse cells. (A) Nano-lantern–labeled Muse cell homing was substantially inhibited by co-injection of S1P receptor 2-specific antagonist JTE-013. (B) Masson trichrome staining of LV in the vehicle, autologous-Muse cells, -non-Muse cells, and -MSC groups at 2 months. Substantial reduction of infarct size and improvement in cardiac function (LVEF) was recognized at 2 months. Figures were redrawn from Yamada et al^15^ with permission. *P < .05, **P < .01, ***P < .001. (**C**) The first-in-human clinical trial of donor Muse cells in AMI.^25^ Changes in LVEF and wall motion score index before and after (until 12 weeks) the administration of CL2020. *P < .05, ***P < .001. Figures were redrawn from Noda et al^25^ with permission. (**D**) Col17-KO EB model mouse that received human Muse cells and CL2020 by intravenous injection. Col17-KO mouse received a single dose of CL2020 and skin samples were collected 4 weeks later. A linear deposition of hCOL7 and hCOL17 in the BMZ (arrowheads) was recognized. Bars. 100 mm. Figures were redrawn from Fujita et al^18^ with permission. (**E-F**) Clinical trial in EB representative clinical images of the patient (patient 1, right lower leg). The area of the erosion rapidly improved after CL2020 administration. Change in the combined size of selected ulcers from the baseline (cm^2^) per patient. *P < .05. Figures were redrawn from Fujita et al^26^ with permission.

Because most AMI patients are treated with PCI to reperfuse the occluded coronary artery, we used AMI models with coronary occlusion and reperfusion in rabbits and mini-pigs. The rabbit coronary artery was occluded for 30 minutes, followed by reperfusion to generate an LV infarction with an area of ~30%.^15^ Twenty-four hours later, when the LV infarction became irreversible, the animals were given intravenous injections of 3 × 10^5^ autologous/allogeneic-Muse cells, -non-Muse cells, or -BM-MSCs. The vehicle group received 2 mL of saline. A comparison of Muse and -non-Muse cells labeled by the luminescent protein Nano-lantern in an in vivo-dynamics analysis revealed that ~14.5% of injected Muse cells homed to the infarcted heart, whereas the non-Muse cells were under the detection limit in the infarcted heart at 3 days (Fig. 3A). Co-injection of Muse cells with an S1P receptor 2 antagonist significantly attenuated the specific homing to the infarcted heart, indicating that the S1P-S1P receptor 2 axis is the main system that controls Muse cell migration and homing (Fig. 3A). Compared with the vehicle group, the non-Muse and MSC groups showed significantly better recovery as evidenced by a reduction of infarct size, improvement of LV function, and attenuation of LV remodeling. The Muse group, however, exhibited significantly better recovery in these parameters than either the non-Muse and MSC groups at 2 months (Fig. 3B). Engrafted Muse cells differentiated into 2 cell types: (1) cardiac cells positive for atrial natriuretic peptides, troponin I, α-actinin, and connexin-43 (Supplementary Fig. S1A) exhibiting Ca^2^ + influx and efflux synchronous with systole and diastole (Supplementary Fig. S1B); and (2) vascular endothelial cells positive for CD31 and α-smooth muscle actin (Supplementary Fig. S1C). The differentiation into cardiac cells was not based on cell fusion, as shown by the fluorescence in situ hybridization experiment, but was initiated by phagocytosing apoptotic-cardiac cell fragments.^12,15^ Human-Muse cells newly expressed cardiac markers after phagocytosing mouse-apoptotic cardiac fragments (Fig. 1E) and were able to show intracellular Ca2 + influx following the biochemical depolarization (Fig. 1F).^1^ Muse cells, non-Muse cells, and MSCs all delivered bystander effects by producing tissue-protective cytokines (ie, HGF and vascular endothelial growth factor) and MMPs, which have anti-fibrosis effects.^15^

Intravenously injected human xenograft-Muse cells also differentiated into cardiac cells and delivered recovery of cardiac function without immunosuppressant treatment at 2 weeks (Supplementary Fig. S1D).^15^ Notably, both the survival of allogeneic-Muse cells as cardiac cells and therapeutic effects lasted up to 6 months without immunosuppressant treatment (Supplementary Fig. S1E).^15^

A loss-of-function experiment using suicide gene (HSVtk)-introduced Muse cells demonstrated that the therapeutic effects, such as a reduction of the infarct size and recovery of LVEF, were significantly attenuated compared with naïve Muse cells at 2 weeks.^15^

A similar trend was confirmed in the mini-pig AMI model in which a semi-clinical grade human Muse cell product was administered without an immunosuppressant. At 2 weeks, Muse cells had differentiated into cardiac and vascular cells, cardiac function improved, and the infarct size was reduced; moreover, no arrhythmias or blood test abnormalities were detected.^36^

These studies suggest that the primary effect of MSCs and non-Muse cells were due to bystander effects, and that the effect of Muse cells was due to their specific homing to the post-infarct heart followed by differentiation into cardiac and vascular cells, in addition to the bystander effects. Furthermore, in contrast to non-Muse cells, which did not remain in the post-infarct heart after 2 weeks, Muse cells, particularly allogeneic-Muse cells, survived in the host cardiac tissue for up to 6 months without immunosuppression.

### A First-In-Human Clinical Trial of Muse Cells for AMI

A first-in-human open-label, non-randomized, single-arm, non-controlled clinical trial using CL2020 was conducted to treat patients with STEMI.^25^ Three STEMI patients with LVEF ≤ 45% after successful PCI were given an intravenous drip of CL2020 containing 1.5 × 10^7^ Muse cells until 5 days after onset. For administration, CL2020 (15 mL) was first diluted with 37 mL of Ringer’s acetate solution, and a total of 52 mL of CL2020 was intravenously infused over 10 ~ 15 minutes. Vital signs were measured, and a medical examination, infectious disease test, hematologic test, blood biochemical test, urinary test, and pro-inflammatory cytokine test (IL-1, IL-6, tumor necrosis factor-alpha, and interferon-gamma measurement at 1, 4, and 12 weeks) were performed consecutively before and after the administration of CL2020 for up to 12 weeks. No blood test abnormalities were associated with the administration of CL2020. No side effects related to Muse cell treatment were detected. LVEF was measured by echocardiography with the modified Simpson’s method before and at 24 hours, 8 days, and 2, 4, 8, and 12 weeks after the administration of CL2020. The LVEF (40.7 ± 1.5% before administration) significantly increased to 43.3 ± 2.1% at 24 hours (*P < *.05), 47.3 ± 3.2% at 8 days (*P < *.001), 49.3 ± 3.2% at 2 weeks (*P < *.001), 47.7 ± 3.2% at 4 weeks (*P < *.001), 50.0 ± 3.0% at 8 weeks (*P < *.001), and 52.0 ± 2.6% at 12 weeks (*P < *.001), for a total LVEF increase from 40.7% to 52.0% with CL2020 treatment over 12 weeks (Fig. 3C).^25^

The significant increase in the LVEF of more than 10% in this clinical trial may improve the long-term prognosis of AMI because the degree of LVEF recovery is reported to predict the long-term prognosis; AMI patients with no LVEF recovery (*Δ* ≤ 0) within 8 weeks have a higher risk of sudden cardiac arrest and death compared with those having modest increase (*Δ*1%-9%) or a significant increase (*Δ* ≥ 10%) in LVEF during a 5-year follow-up.^32^ A substantial increase in LVEF (*Δ* ≥ 10%) is associated with a lower incidence of sudden cardiac arrest and death.^32^

## Epidermolysis Bullosa (EB)

### Cell Therapy for EB

EB is a group of genodermatoses characterized by widespread blisters and erosions caused by mutations in the basement membrane zone (BMZ) genes.^37^ EB is roughly categorized into EB simplex (EBS), junctional EB (JEB), dystrophic EB (DEB), and Kindler’s syndrome according to the cleavage level of the blister observed in ultrastructural studies.^38^ Various structural proteins form the BMZ and as many as 16 causative genes have been identified.^38^ Fundamental to EB is the recurrent injury of the skin and mucous membranes throughout the body. Such damage can induce systemic inflammation with increases in various cytokines, such as IL-6, IL-1β, and IL-12, which results in recurrent fever, fatigue, secondary anemia, and pseudosyndactyly in severe EB patients.^39,40^ Autoimmunity against skin components such as BP180, BP230, and type VII collagen can also be induced in some cases.^39^

Based on these disease characteristics, the following factors must be considered with regard to cell therapies for EB. (1) The transplanted cells must differentiate into keratinocytes (or fibroblasts for DEB). (2) The transplanted cells must retain normal genes encoding basement membrane proteins. (3) Safety must be warranted, particularly in genetics and tumorigenicity. (4) The treatment must be inexpensive, easy to prepare, simple to administer, not rejected by the recipient’s immune system, and repeatable.

Several clinical trials have used bulk MSCs and MSC-based cells, such as ATP-binding cassette subfamily member 5 (ABCB5)-positive mesenchymal cells, for DEB.^18,41-44^ In these studies, most patients showed clinical improvements such as reductions in blister formation, pain, and itch for several months, and amelioration in systemic inflammation by laboratory tests. Due to the rarity of EB in humans, studies examining which stem cells induce the best clinical improvement have not been performed.

In vitro, Muse cells differentiate into keratinocytes, fibroblasts, and melanocytes under specific culture conditions without gene manipulation.^6,45,46^ In vivo, locally injected Muse cells differentiated into keratinocytes and vascular endothelial cells and accelerated wound healing in mice with diabetic skin ulcers compared with non-Muse MSCs.^47^ More recently, a murine atopic dermatitis model generated by repeated application of 2,4-dinitrofluorobenzene was successfully treated by a single subcutaneous injection of Muse cells; the suppression of scratching behavior and decreased expression of pro-inflammatory/itch-related cytokines such as IL-17α, IL-6 IL-33, and IL-1β in the lesional skin were observed.^48^ Based on these observations, the reparative mechanism of Muse cells and non-Muse cells was examined in an animal model of EB.

### Preclinical Studies of Muse Cells in a Mouse EB Model

Several studies investigated the preclinical feasibility of MSCs and their subpopulations for EB by using DEB model mice that are lacking type VII collagen, most of which can survive only several days.^49,50^ Some groups conducted skin grafting onto the back of DEB model mice followed by cell therapies, however, these investigations could not evaluate the survival.^51,52^ Another method to overcome the short life span was to use type XVII collagen-knockout (Col17-KO) mice, which simulate JEB and recurrent skin injuries with long survival.^53,54^ This model is appropriate for evaluating treatments for EB because few EB models can survive for an extended period.^53,54^ Adult Col17-KO mice received 5.0 × 10^4^ Nano-lantern labeled human Muse and non-Muse cells 3 times a week every 2 weeks without immunosuppressant treatment.^18^ Muse cells more effectively homed to skin injury sites than non-Muse cells (Supplementary Fig. S2A). Muse cells that homed to the injured area differentiated into keratin14(+)/human desmoglein-3(+) keratinocytes in the epidermis, keratin 15(+) cells in hair follicles, and CD31(+) vascular endothelial cells in vessels. Muse cells also differentiated into sebaceous glands (Supplementary Fig. S2B). Notably, all the mice that received Muse cells showed a linear deposition of human-type VII COL (hCOL7) and -Col17 in the BMZ of the injury site.^18^

Col17-KO mice were also given a single intravenous injection of 3 × 10^5^ cells/mouse (corresponding to 3 × 10^7^/kg) CL2020 or vehicle without immunosuppressant treatment.^18^ The CL2020-treated mice showed more rapid wound healing, slower expansion of the affected area without genetic mutations, and deposition of hCOL7 and hCOL17 in the BMZ for more than 6 months compared with the vehicle group (Fig. 3D, Supplementary Fig. S2C and S2D).^18^ Interestingly, hair loss and the development of gray hair seemed to be substantially suppressed in the CL2020-treated mice compared with the vehicle group (Supplementary Fig. S2C).^18^

These results suggest that Muse cells are an appropriate therapeutic cell source for treating EB, particularly for COL17 mutated-JEB and DEB. The ability of Muse cells to migrate preferentially to the injured site and replace the diseased epidermal and dermal cells with donor-derived cells differed from that of non-Muse cells. Furthermore, Muse cells did not induce genetic mutations and survived in the host skin for 6 months.

### Clinical Trial of Muse Cells for EB

A phase I/II open-label, non-randomized, single-arm, non-controlled clinical trial of CL2020 in human EB patients was conducted.^26^ The study’s primary endpoint was safety for up to 12 weeks after CL2020 administration. An important secondary endpoint was the ulcer area reduction rate per patient at 4 weeks after CL2020 administration compared with the baseline. Five adult DEB patients (1 man and 4 women, 26.8 ± 12.8 years old) with 13 refractory/recurrent ulcers were enrolled. A single dose of CL2020 containing 1.5 × 10^7^ cells (2.98 ± 0.61 × 10^5^ cells/kg) was intravenously administered, and patients were followed for 52 weeks. Of the 5 EB patients, 2 showed a > 50% reduction in the area of the selected ulcer at 4 weeks after treatment (Fig. 3E, 3F). The combined size of the preselected ulcers was significantly reduced during the 52-week observation period (Fig. 3F). Reduced pain in the ulcer area and improved serum liver enzymes were also observed, possibly due to the anti-inflammatory effect of CL2020. All 5 cases showed mild adverse events within 12 weeks after CL2020 administration, and acquired lacrimal stricture (Grade 2), fever (G1), gastroenteritis (G1), upper respiratory tract infection (G1), and paresthesia of the upper arms (G1) were recorded. The paresthesia, which was considered to be a possible CL2020-related side effect, appeared within 24 hours after the CL2020 administration and resolved within 14 days.^26^ CL2020 might be a potential treatment for adult EB patients, particularly those with DEB. Because this trial was based on a single dose, further studies of the safety and therapeutic effects of multiple doses are needed.

## Ischemic Stroke

### Cell Therapy for Stroke

Stroke is the second most common cause of death and disability worldwide.^55^ Among stroke subtypes, the incidence of ischemic stroke is approximately 80% of total cases, resulting from the shortage of cerebral blood flow due to embolism, atherothrombosis, or small vessel diseases.^55^ Recently, super-acute revascularization therapy, including intravenous administration of recombinant tissue plasminogen activator and mechanical thrombectomy, was developed, and successfully treated patients showed dramatic recovery. Unfortunately, however, these revascularization therapies apply to less than 10% of stroke patients because of the short therapeutic time window.^55^ Those without revascularization therapy develop brain infarction, and more than half of the patients become disabled. Creating a new strategy to promote functional recovery and restore the lost neurological circuit through cell therapy is imperative.

While the exact mechanism by which stem cells repair the damaged brain is not yet fully clarified, the benefits of cell therapy may come from neural circuit reconstruction by direct cell replacement, promotion of endogenous neurogenesis, immunomodulatory effects, neuroprotection, angiogenesis, mitochondria transfer, and/or extracellular vesicle transfer.^56,57^ Various clinical trials using stem cells have been conducted. Most of these trials have demonstrated the safety of the therapy, but the efficacy has not yet been fully established. This may be partly due to the fact that not many studies with a high evidence level, such as double-blinded and placebo-controlled trials, have been conducted. To date, 9 randomized, double-blinded, or observer-blinded trials have been reported.^27,58-65^ Among the 9 studies, 4 used autologous BM-MNCs or BM-MSCs,^59,61,62,64^ 1 used allogeneic BM-derived cells,^60^ 1 used allogeneic MSCs originating from adipose tissue,^65^ 2 used CD34-positive cells or endothelial progenitor cells,^58,63^ and 1 used Muse cells.^27^ An intravenous delivery route was used in 6 studies,^27,59,60,63-65^ an intra-arterial route in 2,^61,62^ and an intracerebral route in 1.^58^ Of the 9 studies, 5 demonstrated statistically significant improvement of some endpoints of functional outcomes.^27,58,60,63,64^ Thus, the effectiveness of stem cell therapy for ischemic stroke was recently investigated in trials with a high level of evidence. For treatment timing, the recent main focus has been allogeneic transplantation in the hyperacute phase, which is expected to deliver neurotrophic factors. On the other hand, autologous transplantation is more focused on the chronic phase, aiming to provide neural replenishment. Although little progress has been made toward developing treatments for the subacute stage, we conducted preclinical studies and clinical trials to assess the safety and reparability of Muse cells, which would allow treatment to be initiated in the subacute phase.

### Preclinical Study of Muse Cells in a Rodent Stroke Model

In rodent stroke models, stereotaxically infused Muse cells successfully integrated into the peri-infarct tissue and expressed neuronal and oligodendrocyte markers, while few non-Muse cells integrated into the peri-infarct tissue.^66^ Injected human Muse cells took ~3 days to express early neural markers, Mash1 and NeuroD, and ~7 days to express maturity markers, MAP2 and NeuN, after the injection, making connections with each other in the homed post-infarct tissue in the rat stroke model.^66,67^ The human Muse cell-derived neuronal cells were further incorporated into the pyramidal tract, including the pyramidal decussation, as demonstrated by retrograde and anterograde tracing (Supplementary Fig. S3A–S3C).^66,67^ Similarly, they were incorporated into the sensory tracts, as shown by the recovery of somatosensory-evoked potentials and the formation of synapses with host neuronal cells (Supplementary Fig. S3D–S3F). The non-Muse and MSC groups tended to exhibit better motor (modified neurological severity score and rotarod, corner turn, and cylinder tests) and sensory function scores than the vehicle group, whereas the Muse group exhibited significantly meaningful therapeutic effects in these assessments compared with the non-Muse, MSC, and vehicle groups within 3 months.^66,67^

**Figure 4.**
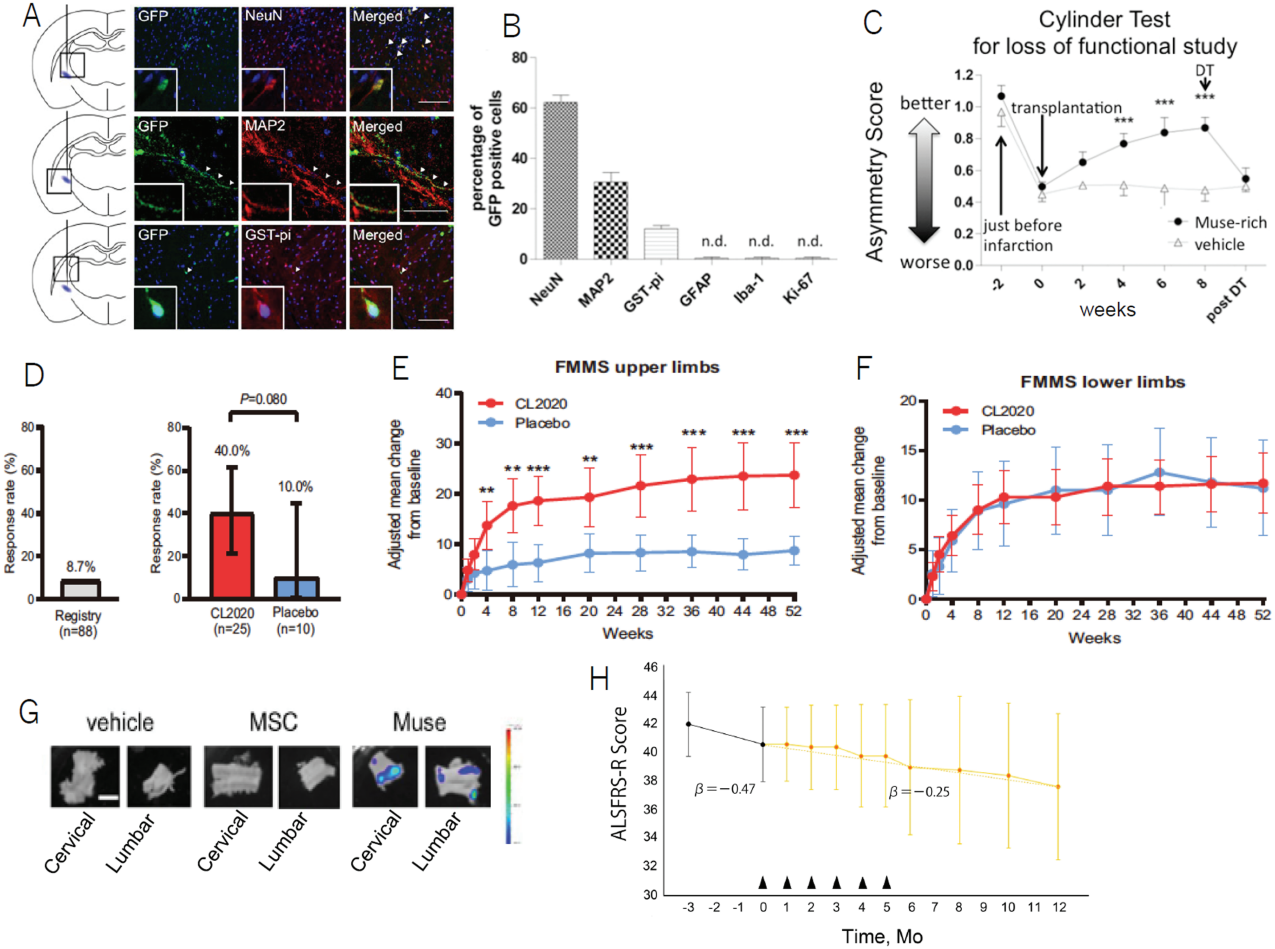
Preclinical and clinical trials in stroke and ALS. (**A-B**) Immunodeficient mouse with lacunar infarction received human Muse cell injection at 2 weeks.^67^ Differentiation of GFP-Muse cells into neuronal (NeuN, MAP2)-, oligodendrocyte (GST-pi)-, astroglial (GFAP)-, microglial (Iba-1)-, and proliferative cell (ki-67)-markers. (**C**) Loss of function experiment by intraperitoneal injection of diphtheria toxin (DT). Figures A-C were redrawn from Uchida et al, 2017^67^ with permission. (**D-F**) Clinical trial in subacute stroke.^27^ (D) Responder analysis of patients with modified Rankin Scale (mRS) scores of 0-2 at 12 weeks, demonstrating mean (95% CI) response rates in the CL2020 (40.0% [21.1, 61.3]) and placebo (10.0% [0.3, 44.5]) groups; the threshold response rate of 8.7% based on registry data. The lower limit of the 95% CI in the CL2020 group was higher than the 8.7% seen in the registry data. The difference between the CL2020 and placebo groups was analyzed by Fisher’s exact test (mid-*P* value). (**E**) Adjusted mean (95% CI) change from baseline in Fugl–Meyer Motor Scale (FMMS) upper limb score, and (**F**) lower limb score in the CL2020 (*n* = 19-25) and placebo (*n* = 8–10) groups. **P < .01, ***P < .001. Figures were redrawn from Niizuma et al^27^ with permission. (**G**) Mouse ALS model that received intravenous injection of human Muse cells.^75^ Distribution of nano-lantern-human MSCs and Muse cells in the cervical and lumbar spinal cord 7 days after intravenous injection. Figures were redrawn from Yamashita et al^75^ with permission. (**H**) Clinical trial in ALS.^28^ Longitudinal follow-up of the changes in the ALSFRS-R scores. Mean (SD) ALSFRS-R scores of patients with ALS treated with CL2020. Arrowheads indicate the intravenous administration of CL2020. ALSFRS-R, Revised Amyotrophic Lateral Sclerosis Functional Rating Scale; mo, months. Figures were redrawn from Yamashita et al^28^ with permission.

Integrated human Muse cells, injected both at the acute phase (2 days after onset) and the subacute phase (2 weeks after onset), differentiated into cells positive for neuronal markers (NeuN and MAP2) and glial cells (GST-pi) in the post-infarct tissue (Fig. 4A, 4B). When integrated Muse cells were eliminated by a loss of function experiment at 8 weeks, the time at which functional recovery was stable in the cylinder test compared with the vehicle group, active recovery was abolished and dropped sharply to the vehicle group level, suggesting that integrated Muse cells mediated the behavioral outcome (Fig. 4C).^67^ Tumor formation was not identified in the brain, lung, kidney, liver, or spleen, and Muse cells did not disseminate to other tissues for up to 10 months.^66,67^

A similar trend was observed following intravenous injection of CL2020 into a mouse lacunar stroke model at days 9 (subacute phase) and 30 (chronic phase).^16^ The extent of functional recovery was dose-dependent and effective even when the injection was administered on day 30. Persistent therapeutic effects were confirmed for up to 22 weeks without outstanding adverse effects.^16^ Notably, intravenously injected Muse cells were observed to reach the lacunar infarction area in 1 day (Supplementary Fig. S3G).^16^

These results suggest that while non-Muse cells and MSCs have some therapeutic effects, Muse cells had stronger therapeutic effects than either non-Muse cells and MSCs, partly due to the replacement of neural cells and reconstruction of neuronal circuits.

### Clinical Trial of Muse Cells for Subacute Stroke

A double-blind, placebo-controlled clinical trial enrolled ischemic stroke patients with a modified Rankin Scale (mRS) ≥ 3.^27^ Randomized patients were given a single dose intravenous drip of either CL2020 (*n* = 25) or placebo (*n* = 10) without immunosuppressant 14-28 days after stroke onset. The safety (primary endpoint: 12 weeks) and efficacy (mRS, other stroke-specific measures) were assessed for up to 52 weeks. The key efficacy endpoint was the response rate (percentage of patients with mRS ≤ 2 at 12 weeks). At 12 weeks, adverse reactions were experienced in 28% of the CL2020 group (including one Grade 4 status epilepticus) and 10% of the placebo group. Although the study was conducted without immunosuppressants, no serious adverse reactions attributable to rejection were observed during the 52-week follow-up. The response rate was 40.0% (95% CI, 21.1, 61.3) in the CL2020 group and 10.0% (0.3, 44.5) in the placebo group; the lower CI in the CL2020 group exceeded the preset efficacy threshold (8.7% from registry data; Fig. 4D).^27^

Moreover, significant improvements in the Fugl–Meyer Motor Scale upper limb and total scores were observed as early as 4 weeks and continued to 52 weeks.^27^ Such early improvements in upper limb function are difficult to achieve and are directly related to improving mRS to 0 or 1. Indeed, previous research suggests that upper limb function is essential to “dressing” (changing clothes) as an activity of daily living and is therefore highly correlated with patients becoming independent (Fig. 4E–4F).^68^ Because no cell tracking was performed in the clinical trial, it is unclear whether or not the Muse cells maintained engraftment for a long period. It is also unclear how the Muse cells exerted their therapeutic effect on patients. Multiple mechanisms, such as neurotrophic factors, direct engraftment, or activation of endogenous regeneration, are possible, and further trials are needed.

Interestingly, in the CL2020 group, 3 (12%) patients experienced a change in hair color from gray/white to black within 12 weeks, and 3 additional patients experienced a difference in hair color between 12 weeks and 52 weeks, for a total of 6 patients (24%); no patients in the placebo group experienced a change in hair color.^27^ Unexpectedly, the Muse cell therapy effects are suggested to extend to areas related to hair color in addition to stroke.

In conclusion, Muse cells demonstrated promising treatment effectiveness in animal and clinical studies. They may repair injured neuronal tissue mainly by cell replenishment. In the clinical trial, Muse cells rapidly improved upper limb function, which might contribute to more remarkable functional recovery, and no significant safety issues were observed. Muse cells are promising treatments for ischemic stroke and may enhance the accessibility to regenerative medicine.

## Amyotrophic Lateral Sclerosis (ALS)

### Cell Therapy for ALS

Cell therapies for ALS have been developed by several researchers, primarily targeting the regeneration of nerve and muscle cells, suppression of inflammation, and neuroprotection.^69^ BM-MSCs have demonstrated therapeutic potential for neurodegenerative diseases, including ALS.^3^ Recently, clinical trials were conducted with MSCs modified to secrete neurotrophic factors (called MSC-NTF). The phase II trial, which enrolled 48 ALS patients who were randomized 3:1 (autologous bone marrow-derived MSCs-NTF: placebo), demonstrated that the rate of disease progression was improved at early time points.^70^ The phase III trial, however, showed no statistically significant difference between the MSCs-NTF and placebo groups in the proportion of responders as the primary endpoint or in the progress of the ALSFRS-R as the secondary endpoint.^71^

Neural stem cells (NSCs) are considered another potential cellular resource for ALS therapy. Early preclinical studies evaluated the therapeutic potential of established NSCs in SOD1-G93A transgenic rats, demonstrating that transplantation of NSCs improves motor function and prolongs life span.^72^ Notably, Glass et al^73^ performed an open-label clinical trial of spinal cord transplantation of human NSCs in ALS patients. All participants were given bilateral injections of NSCs (0.5-16 million) into the cervical spinal cord (C3-C5). This study showed that intraspinal transplantation of human spinal cord-derived NSCs could be safely accomplished even at high doses, but the therapeutic effect was unclear.^73^

ALS patients have difficulty maintaining their posture due to limb muscle weakness; and thus intrathecal injection is burdensome. As Muse cells can selectively reach the site of damage following intravenous drip, they are expected to be advantageous for treating ALS patients.

### Preclinical Study of Muse Cells in a Mouse ALS Model

The therapeutic potential of human MSCs and Muse cells was evaluated in SOD1-G93A transgenic mice expressing the G93A mutant form human of SOD1, which exhibits a phenotype similar to that of ALS patients.^74^ The homing of Muse cells to the spinal cord was compared between intravenous and intrathecal injections (Supplementary Fig. S4A).^75^ Intravenous injection was more efficient than intrathecal injection in delivering Muse cells to the cervical and lumbar spinal cord, an important therapeutic target of ALS (Fig. 4G). MSCs were under the detection limit in the cervical and lumbar spinal cord (Fig. 4G). The therapeutic effects were compared among human Muse cells, MSCs, and vehicle groups based on behavioral test scores, enhanced motor neuron survival, and reduced myofiber atrophy. The Muse cell group showed statistically significant therapeutic effects in these parameters at various time points during the 154 observation days (Supplementary Fig. S4B–S4D). While the MSC group showed an increase in motor neuron survival in the lumbar spinal cord, amelioration of atrophy, and neuromuscular junctions in the tibialis anterior muscle compared with the vehicle group, the Muse group was significantly superior to the MSC and vehicle groups in these assessments (Supplementary Fig. S4E, S4F).^75^

These findings suggest that Muse cells, which can reach the target spinal cord by intravenous injection and deliver some of the therapeutic effects, are a potential treatment option for ALS.

### Clinical Trial of Muse Cells for ALS

Unlike in the clinical trials for AMI and ischemic stroke, in which tissue damage occurs suddenly, CL2020 was administered multiple times for patients with ALS, in which motor neuron degeneration progresses slowly and continuously. To confirm the safety of multiple intravenous injections of CL2020, 5 patients with sporadic ALS (male:female ratio of 3:2) with the limb-onset clinical form were enrolled in an open-label, non-randomized, single-arm, non-controlled clinical trial.^28^ Patients were given intravenous injections of CL2020 once a month for 6 months. The primary endpoints were safety and tolerability, and the secondary endpoint was the rate of change in the Revised Amyotrophic Lateral Sclerosis Functional Rating Scale (ALSFRS-R) score until 12 months.

Despite repeated administration of CL2020 prepared from donors without HLA-matching tests or immunosuppressant treatment, the treatment was highly tolerated, with no incidence of pulmonary embolisms, anaphylactic shock, or other serious adverse effects. Common adverse events were headaches and fatigue. Whether or not the 6 injections were derived from single or multiple donors is unknown.

Notably, the ALSFRS-R scores remained unchanged, that is, they did not progress, in 4 patients. Among these 4 patients, 1 showed a trend toward exacerbation for up to 10 months after initiating cell administration (Fig. 4H). These results strongly suggest the therapeutic potential of CL2020 treatment for mitigating ALS progression. Unfortunately, 12 months after the first dose, patient #2 fractured the proximal left humerus and left ankle joint during a fall. This incidental adverse event resulted in a worsening of clinical scores at 12 months, including the ALSFRS-R (Fig. 4H). As a result, although the ALSFRS-R scores tended to improve 12 months post-treatment compared with 3 months before CL2020 administration, the difference did not reach statistical significance, likely due to the incident experienced by patient #2.

In addition, the serum S1P level, relevant to the pathogenesis of neurodegenerative diseases including ALS and the factor that is known to mediate Muse cell migration,^76,77^ continuously decreased over 12 months. Considering that fingolimod, a functional S1P antagonist, exerted beneficial effects through modulation of the immune response in an ALS mouse model,^78^ the administered CL2020 might have functionally neutralized S1P, leading to the attenuation of S1P-related inflammatory signaling in ALS.

These findings suggest a favorable safety profile for CL2020 therapy and indicate the potential of the treatment to reduce pathologic changes in ALS. The small number of patients (*n* = 5) and the lack of comparators, however, limit the interpretation of the study results. Therefore, a double-blind study with more ALS patients is needed to confirm the efficacy of CL2020 therapy.

## Future Perspectives

Generally, donor-derived organ and BM transplantation require long-term immunosuppressant treatment, which may increase the infection risk. When allogeneic cells are infused or transplanted in vivo without HLA matching or immunosuppressants, cells are quickly rejected by the host’s immune system; BM cells from BALB/c mice transplanted into C57BL/6 mice were rejected within 3 hours in the earliest cases.^79^ The fact that donor Muse cell treatment can be administered without the need for immunosuppressants is of extraordinary significance and a great advantage for clinical applications. Furthermore, donor cells may be more valuable than autologous cells because they are ready to use and are available during emergencies and acute phases, are less burdensome for the patients than Muse cell collection, and provide ease of quality control. While the Muse cell expression of HLA-G, as well as their production of indoleamine 2, 3-dioxygenase, TGF-β, PGE2, nitric oxide, and HGF are suggested to be involved in suppressing the host immune system,^2,15,17^ it remains unclear how the long-term survival of HLA-mismatched Muse cells is achieved and this is an important point that must be clarified in future studies.

The loss-of-function experiment in the AMI rabbit and lacunar stroke mouse models revealed that eliminating Muse cells that were once integrated as cardiac cells and neural cells, respectively, rapidly abolished the functional improvement.^15,67^ In humans as well, the functional improvement established by integrated Muse cells will be diminished or disappear if the Muse cells are rejected by immunologic attack. In long-term observations after the clinical trial, the engraftment or rejection of donor Muse cells might be estimated depending on whether the improvement effect continues to be maintained or the impact diminishes. Sustained improvement may be possible due to the continuous action of donor Muse cells once engrafted. It is also possible, however, that donor Muse cells gradually disappear and endogenous Muse cells, such as in the BM, take over the role of the donor Muse cells to maintain stable, beneficial effects. In this case, a novel strategy would be to treat the acute phase patient with donor Muse cells and then prepare autologous Muse cells for the chronic phase treatment.

The clinical studies also revealed that the optimal conditions for administration timing, number of administrations, and number of donor Muse cells vary depending on the disease. In AMI and stroke, where tissue damage occurs acutely, a single dose appeared to be therapeutically effective.^25,27^ The same trend was confirmed in animal models.^15,16,67^ On the other hand, ALS, which progresses gradually, and EB, which causes life-long skin blistering and peeling, have significantly different pathologies from AMI and stroke. Although with only 5 patients, multiple doses of Muse cells prevented or slowed the deterioration of the disease condition in ALS.^28^ In EB, on the other hand, where only 1 dose was administered in the clinical trial, the improvement continued over a certain period,^26^ but even stronger effects are expected with multiple doses. In chronic diseases, it might be worth investigating whether the engraftment of healthy donor Muse cells into the patient’s BM, the site of the reserve of endogenous Muse cells, to supply them to the area of damage would provide long-term and continuous therapeutic effects.

The brain has a blood-brain barrier and is immunologically different from other organs. The clinical trials in ischemic stroke showed interesting results regarding the optimal timing of administration. Muse cells are known to selectively migrate to damaged tissue by sensing S1P. In stroke patients, however, therapeutic effects were still recognized when donor Muse cells were administered at the phase close to the chronic phase when the S1P signal was substantially decreased compared with that in the acute phase.^27^ This suggests that donor Muse cell treatment can be developed for treating chronic phase stroke patients, which would have a significant social impact.

Conventional MSCs, already widely applied in clinical studies, are usually trapped in the lung capillaries after intravenous injection and do not survive in the body for a long period,^15^ and thus their therapeutic effects are mainly attributed to bystander effects.^1^ Similar to MSCs, Muse cells are capable of providing bystander effects.^15,17,19^ As demonstrated in preclinical studies, however, Muse cells have unique characteristics distinct from MSCs such as selective homing to the target tissue, rapid differentiation into the target cell type to replace damaged/apoptotic cells in the tissue, and survival in the homed tissue for an extended period due to their specific immunotolerance.^2^ Because there was no enrollment of an MSC administration group in the Muse cell clinical studies, the therapeutic effects of Muse cells and MSCs cannot be directly compared. As the comparison between MSCs/non-Muse cells and Muse cells in preclinical studies demonstrated, however, the differences in therapeutic efficacy might be due to differences in the tissue repair mechanisms.

## Limitation of Muse Cells for Clinical Application

Clinical trials for AMI, EB, ischemic stroke, and ALS were all conducted by intravenous injection of an allogeneic-human BM-Muse cell product and followed up for a certain period without immunosuppressant treatment.^25-28^ Although the sample sizes in these trials were small and thus insufficient to draw conclusions, no tumorigenesis or severe side effects that required interruption of the trials were observed during the follow-up. The results of the trials demonstrated the possibility of a therapeutic impact of Muse cells. Further placebo-controlled double-blind studies with large sample sizes and more extended follow-up periods are required to assess the efficacy of Muse cell treatment.

Acute diseases such as AMI and stroke, and chronic diseases such as EB and ALS differ in their disease progression, pathologies, and clinical features. In ALS, multiple doses with proper intervals are expected to be more efficient than a single-dose treatment. In this case, it will be necessary to verify how many donors can be effectively used without causing rejection.

## Supplementary Material

Supplementary material is available at *Stem Cells Translational Medicine* online.

szae018_suppl_Supplementary_Figures_S1-S4

## Data Availability

The data underlying this article will be shared on reasonable request to the corresponding author.
