# Supplementary material for: Donor Muse Cell Treatment Without HLA-Matching Tests and Immunosuppressant Treatment
Source: Stem Cells Transl Med. 2024 Jun 10;13(6):532–45. doi: 10.1093/stcltm/szae018 (PMC11165166; doi:10.1093/stcltm/szae018)
Supplement: szae018_suppl_Supplementary_Figures_S1-S4 [file szae018_suppl_supplementary_figures_s1-s4.docx]

**Donor Muse cell treatment without HLA-matching tests and immunosuppressant treatment**

Supplement Figures


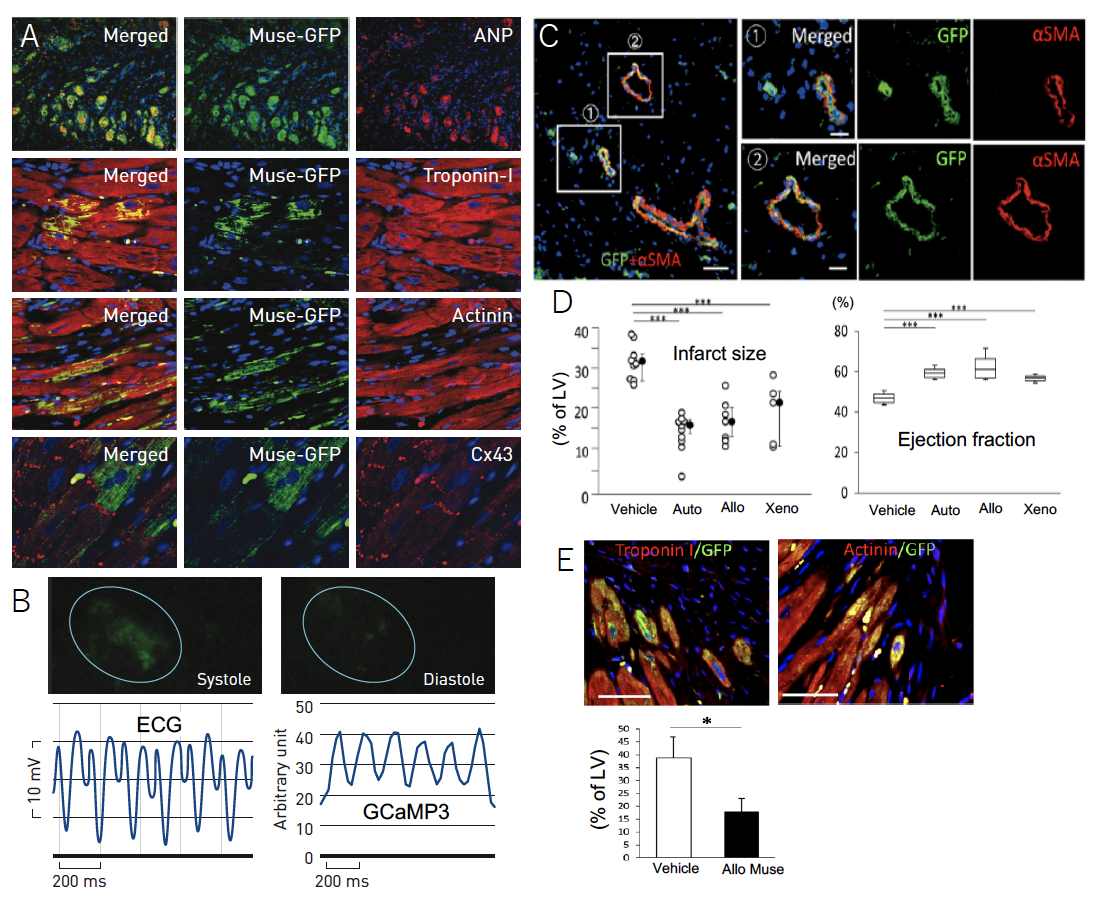


Figure S1. AMI rabbit model that received intravenous injection of Muse cells.^1^

A) Green fluorescent protein (GFP)-labeled Muse cells differentiated into atrial natriuretic peptide (ANP)-, troponin I-. actinin- and connexin-43 (Cx43)-positive cells at 2 weeks in the homed heart tissue. B) In vivo image of GCaMP3 fluorescence in systole and diastole 2 weeks after receiving GCaMP3-Muse cells. Synchronization of electrocardiogram and time-intensity curve of GCaMP is shown. C) Differentiation of GFP-Muse cells into smooth muscle actin (alpha SMA)(+) vascular cells in the heart tissue. D) improvement in the Infarct size reduction and LVEF (ejection fraction) in the vehicle, auto-, allo-, and xeno-Muse cell injection at 2 weeks. E) Allogeneic-GFP-Muse cells remained in the heart tissue as troponin-I (+) and actinin(+) cardiac cells 6 month after intravenous injection. Infarct size was significantly reduced in the allo-Muse cell group compared with the vehicle group. **P*< .05, ***P*< .01, ****P*< .001. Figures were redrawn from Yamada et al^1^ with permission.


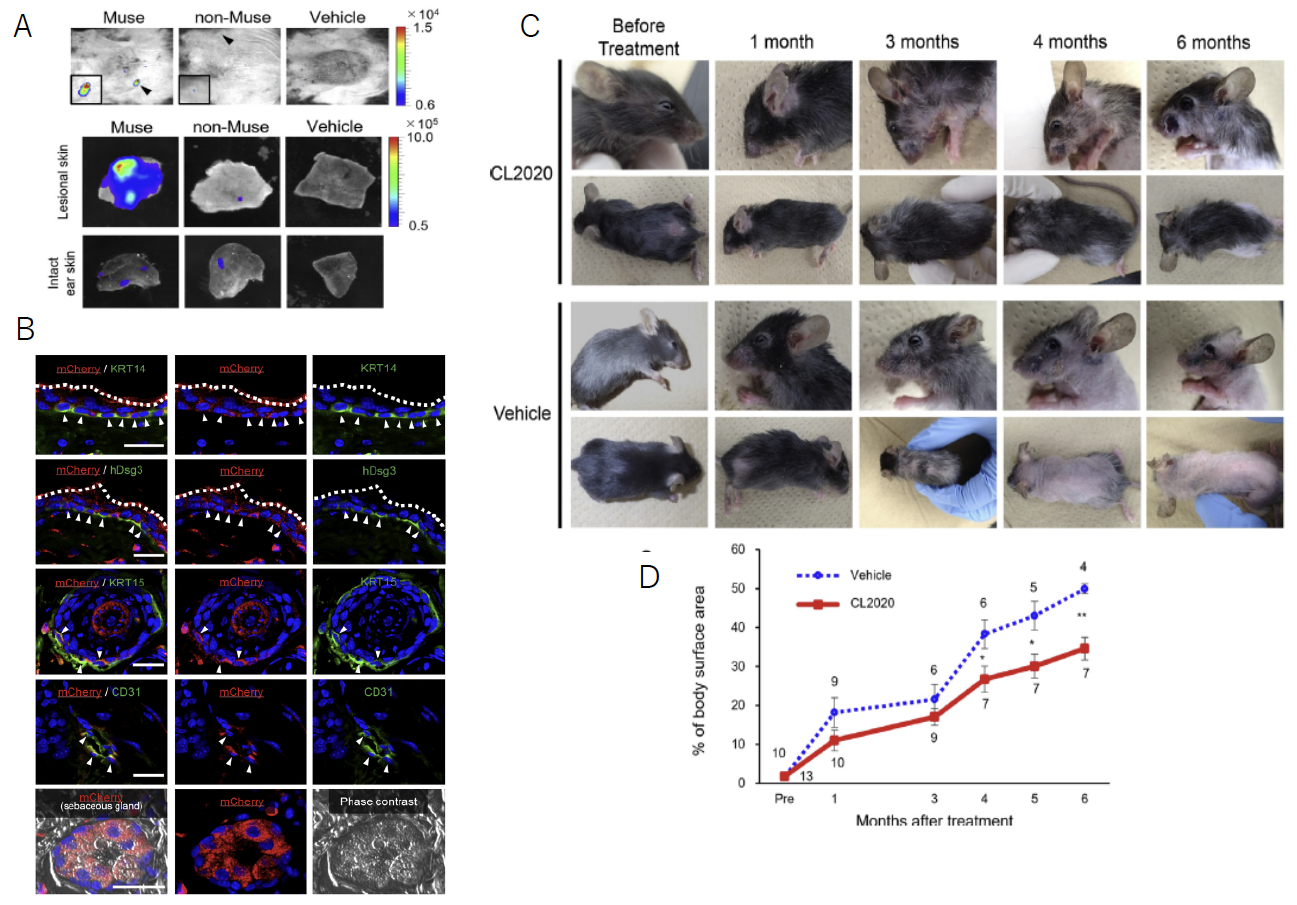


Figure S2. Col17-KO EB model mouse that received human Muse cells and CL2020 by intravenous injection.^2^

A) Mice received an intravenous injection of human Muse cells, non-Muse cells, or vehicle through the tail vein 3 times every 2 weeks without the administration of an immunosuppressant. They were killed at 7 days after the last injection. In vivo imaging shows Nano-lantern-Muse cells, but not non-Muse cells or vehicle, accumulated to the injured site (7 days). B) mCherry-Muse cells differentiated into epidermal keratinocytes, hair follicle cells, vascular endothelial cells, and sebaceous gland cells (arrowheads) (7 days). C) The CL2020-treated mice showed milder skin symptoms than vehicle-treated mice, particularly after 3 months. Notably, hair loss and the development of gray hair was prevented in the CL2020-treated mice. D) The vehicle-treated (control) mice show more severe skin scores than the CL2020-treated mice. Figures were redrawn from Fujita et al^2^ with permission.


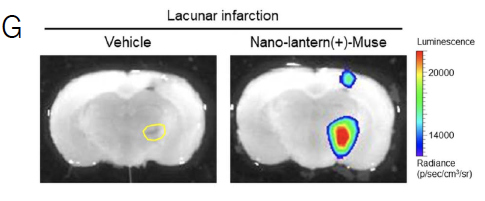

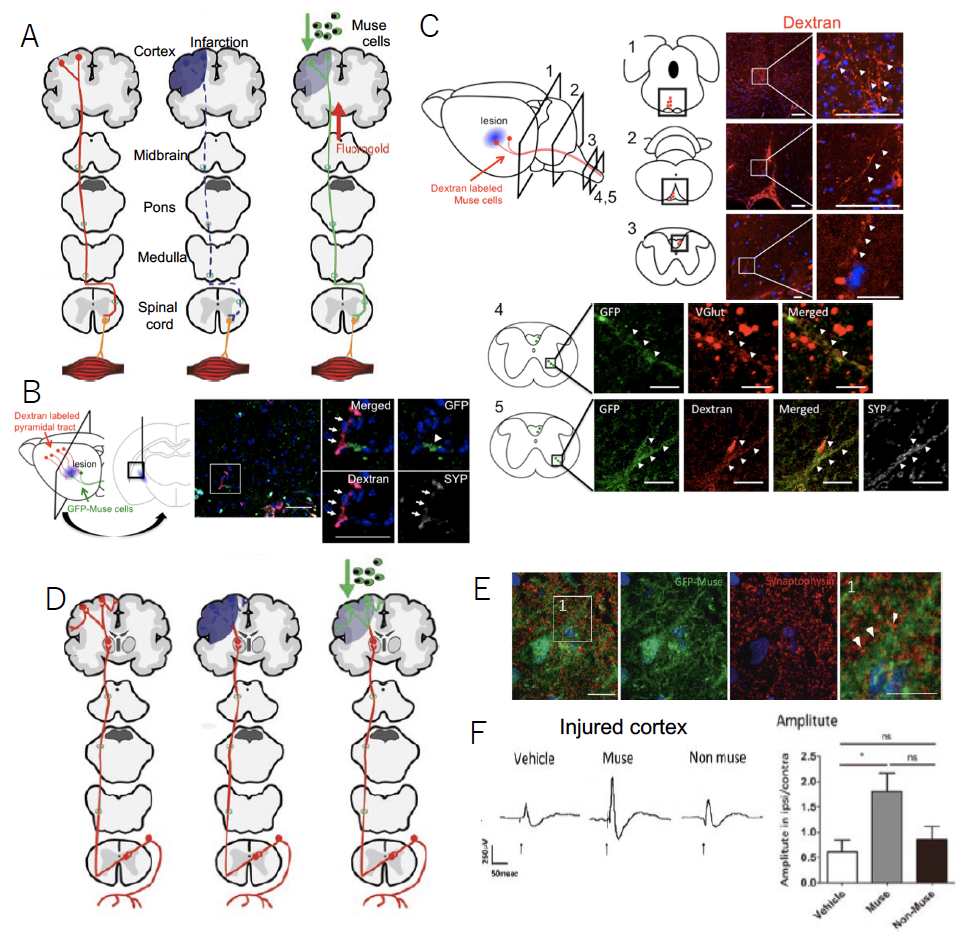


Figure S3. Rodent stroke model that received human Muse cell treatment.^3-5^

A) Schematic figure showing pyramidal tract reconstruction, including the pyramidal decussation. B) Immunodeficient mouse received GFP-human Muse cell injection into the perilesional brain 2 weeks after lacunar infarction.^5^ Motor cortical neurons were anterogradely labeled with dextran. GFP-Muse cells (arrowhead) connected to dextran-labeled motor neuron axons (red, arrows), that merged with synaptophysin (SYP, arrows). C) Dextran-labeled axons(red, arrowheads) were detected at level 1: midbrain and level 2: medulla on the ipsilateral side and in the cervical spinal cord at levels 3 to 5 on the contralateral side 8 weeks after. Levels 4 and 5 show the area of anterior horn in the spinal cord where pyramidal tract axons formed synapses with motor neurons. GFP+ neurite positive for VGlut, a marker for glutamatergic neuron (level 4). Dextran (red)–labeled GFP (green)–positive Muse cells were positive for synaptophysin (white) in the upper cervical spinal cord (level 5).^5^ Scale bars, 100 μm (B), 50 μm (C: levels 1 and 2); and 10 μm (C: levels 3–5). D) Schematic figure showing sensory tract reconstruction. E) Rat middle cerebral artery occlusion model received a stereotaxic injection of human Muse cells at 84 days. In the ipsilateral sensory cortex, synaptophysin (red) was detected adjacent to dendrite-like structure of GFP-labeled Muse cells (green) (arrowheads).^4^ Scale bar=10 μm. F) Somatosensory evoked potentials (SEP) in the vehicle, Muse, and non-Muse groups in the ipsilateral sensory cortex. The graph shows the ratio of SEP amplitude on ipsilateral side to that on contralateral side.^4^ *: *P*< .05. G) Homing of intravenously administered Nano-lantern human Muse cells to the infarct region 1 day after.^3^ Figures were redrawn from Uchida et al., 2016,^4^ Uchida et al., 2017,^5^ and Abe et al, 2020^3^ with permission.


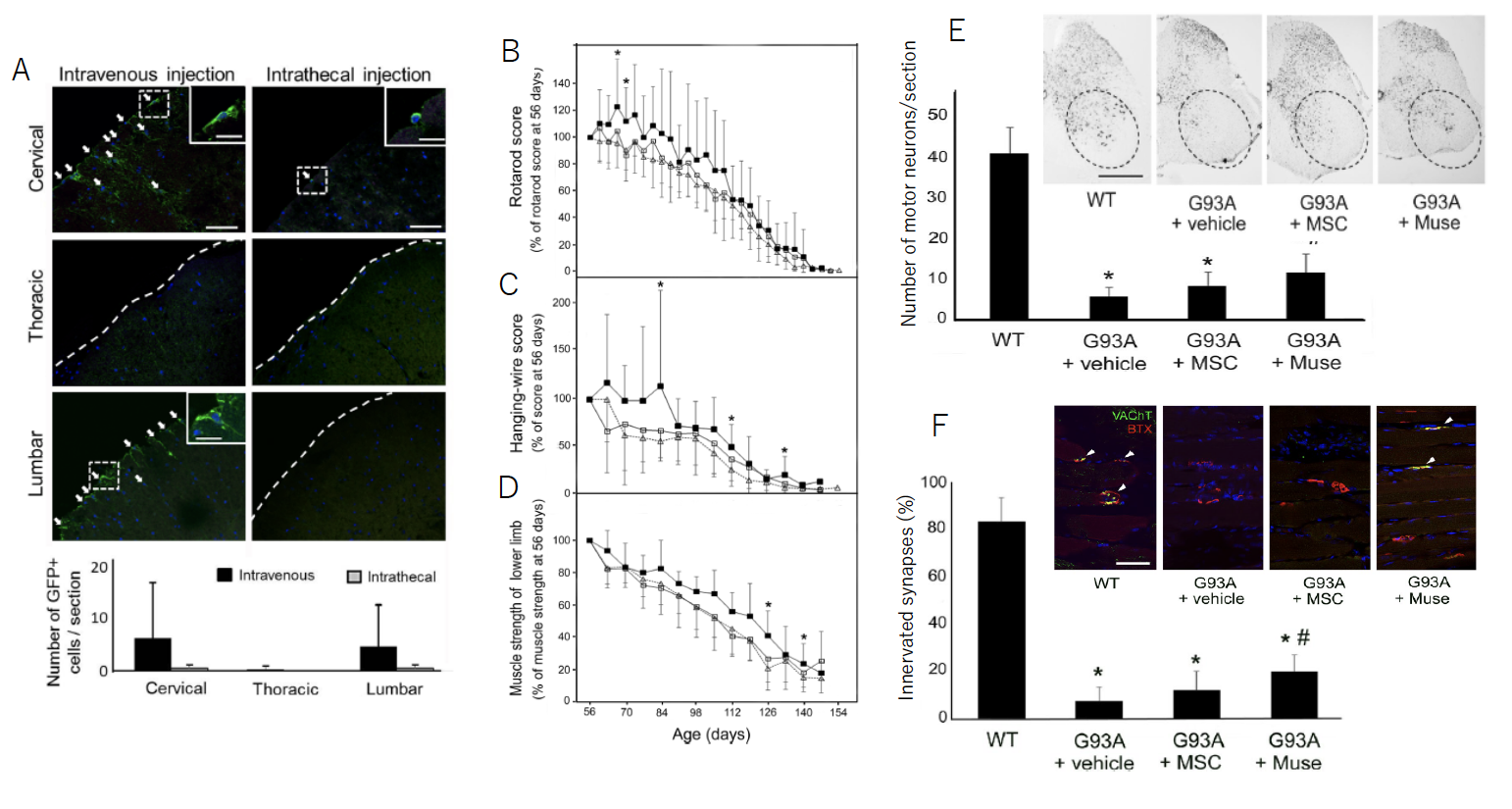


Figure S4. Mouse ALS model that received intravenous injection of human Muse cells.^6^

A) GFP- Muse cells in the spinal cord at 7 days after intravenous (IV) or intrathecal (IT) injection.

Only IV injection delivered many Muse cells to the pia-mater and underneath the white matter of both cervical and lumbar spinal cords. Scale bars: 100 μm (a); 20 μm (a, inset). (B-D) Behavioral analysis in (B) rotarod test, (C) hanging-wire test, and (D) muscle strength of lower limbs. Compared with the vehicle, Muse cells induced significant improvements (**P*< .05, vs vehicle). E) Number of Nissl-stained motor neurons in the lumber spinal cord (**P*< .05, vs wild type = WT)(#*P*< .05, vs vehicle). Scale bar: 500 μm (a). F) Staining of the neuromuscular junction (NMJ) in the tibialis anterior muscle (VAChT-positive motor terminals; green, acetylcholine receptors stained with BTX; red, arrowheads, **P*< .05, vs WT, #*P*< .05, vs vehicle). Scale bar: 50 μm. Figures were redrawn from Yamashita et al^6^ with permission.

**References**

1. Yamada Y, Wakao S, Kushida Y, et al. S1P-S1PR2 Axis Mediates Homing of Muse Cells Into Damaged Heart for Long-Lasting Tissue Repair and Functional Recovery After Acute Myocardial Infarction. *Circ Res* 2018; **122**(8): 1069-83.

2. Fujita Y, Komatsu M, Lee SE, et al. Intravenous Injection of Muse Cells as a Potential Therapeutic Approach for Epidermolysis Bullosa. *J Invest Dermatol* 2021; **141**(1): 198-202 e6.

3. Abe T, Aburakawa D, Niizuma K, et al. Intravenously Transplanted Human Multilineage-Differentiating Stress-Enduring Cells Afford Brain Repair in a Mouse Lacunar Stroke Model. *Stroke* 2020; **51**(2): 601-11.

4. Uchida H, Morita T, Niizuma K, et al. Transplantation of Unique Subpopulation of Fibroblasts, Muse Cells, Ameliorates Experimental Stroke Possibly via Robust Neuronal Differentiation. *Stem Cells* 2016; **34**(1): 160-73.

5. Uchida H, Niizuma K, Kushida Y, et al. Human Muse Cells Reconstruct Neuronal Circuitry in Subacute Lacunar Stroke Model. *Stroke* 2017; **48**(2): 428-35.

6. Yamashita T, Kushida Y, Wakao S, et al. Therapeutic benefit of Muse cells in a mouse model of amyotrophic lateral sclerosis. *Sci Rep* 2020; **10**(1): 17102.
